# Supplementary material for: High Iron Exposure from the Fetal Stage to Adulthood in Mice Alters Lipid Metabolism
Source: Nutrients. 2022 Jun 13;14(12):2451. doi: 10.3390/nu14122451 (PMC9227341; doi:10.3390/nu14122451)
Supplement: Supplementary file 1 [file nutrients-14-02451-s001.zip › nutrients-1713430-supplementary.pdf]

Supplementary Table S1. List of putative transcription factor binding sites detected in mouse FTH1 upstream 1.0-kb predicted with a dissimilarity margin level less or equal than 15% in PROMO ALGGEN.

Factor name	Start position	End position	Dissimilarity	String	RE equally	RE query
AP-1 [T00029]	597	605	5.354713	TGACTCCCA	0.03815	0.02927
AP-1 [T00029]	946	954	13.075412	CCAAAGTCA	0.14496	0.17976
AP-1 [T00032]	597	605	9.544233	TGACTCCCA	0.06104	0.03719
AP-1 [T01140]	508	514	14.285714	TGAGACA	1.28174	1.33865
AP-1 [T01140]	597	603	14.285714	TGACTCC	1.28174	1.33865
C/EBP [T01388]	148	154	1.299659	CTGAGCA	0.48828	0.26141
C/EBP [T01388]	283	289	2.84092	TGCCCAC	0.12207	0.04332
C/EBP [T01388]	328	334	0	TTGTGCA	0.36621	0.31784
C/EBP [T01388]	368	374	1.541262	ATGGGCA	0.48828	0.26141
C/EBP [T01388]	979	985	1.299659	CTGTGCA	0.48828	0.26141
C/EBPα [T00104]	28	31	0	AAAT	3.90625	9.71949
C/EBPα [T00104]	162	165	0	ATTT	3.90625	9.71949
C/EBPα [T00104]	217	220	0	AAAT	3.90625	9.71949
C/EBPα [T00104]	318	321	0	AAAT	3.90625	9.71949
C/EBPα [T00104]	423	426	0	AAAT	3.90625	9.71949
C/EBPα [T00104]	582	585	0	AAAT	3.90625	9.71949
C/EBPα [T00104]	584	587	0	ATTT	3.90625	9.71949
C/EBPα [T00104]	637	640	0	ATTT	3.90625	9.71949
C/EBPα [T00104]	694	697	0	AAAT	3.90625	9.71949
C/EBPα [T00104]	696	699	0	ATTT	3.90625	9.71949
C/EBPα [T00104]	734	737	0	ATTT	3.90625	9.71949
C/EBPα [T00104]	754	757	0	ATTT	3.90625	9.71949

C/EBPα [T00104]	776	779	0	ATTT	3.90625	9.71949
C/EBPα [T00104]	782	785	0	AAAT	3.90625	9.71949
C/EBPα [T00104]	798	801	0	ATTT	3.90625	9.71949
C/EBPα [T00104]	857	860	0	ATTT	3.90625	9.71949
C/EBPα [T00104]	862	865	0	AAAT	3.90625	9.71949
C/EBPα [T00104]	914	917	0	ATTT	3.90625	9.71949
C/EBPα [T00104]	920	923	0	ATTT	3.90625	9.71949
C/EBPα [T00105]	5	10	2.897783	TTGCAA	0.48828	0.52285
C/EBPα [T00105]	363	368	2.443272	TTGCTA	0.97656	1.0805
C/EBPα [T00105]	370	375	0.681767	GGGCAA	0.48828	0.32078
C/EBPα [T00105]	586	591	2.514221	TTGCCT	0.97656	1.0805
C/EBPα [T00107]	470	477	14.367318	TTTGGTTT	0.36621	0.46209
C/EBPα [T00107]	646	653	5.228028	TGGCCAAA	0.06104	0.04527
C/EBPα [T00107]	689	696	10.446297	ATTCCAAA	0.42725	0.36963
C/EBPα [T00107]	697	704	4.011491	TTTGGATA	0.12207	0.20872
C/EBPα [T00107]	943	950	10.446297	GTACCAAA	0.42725	0.36963
C/EBPα [T00108]	5	7	0	TTG	15.625	18.2634
C/EBPα [T00108]	8	10	0	CAA	15.625	18.2634
C/EBPα [T00108]	74	76	0	CAA	15.625	18.2634
C/EBPα [T00108]	78	80	0	CAA	15.625	18.2634
C/EBPα [T00108]	139	141	0	CAA	15.625	18.2634
C/EBPα [T00108]	164	166	0	TTG	15.625	18.2634
C/EBPα [T00108]	259	261	0	CAA	15.625	18.2634
C/EBPα [T00108]	307	309	0	CAA	15.625	18.2634
C/EBPα [T00108]	328	330	0	TTG	15.625	18.2634

C/EBPα [T00108]	350	352	0	TTG	15.625	18.2634
C/EBPα [T00108]	363	365	0	TTG	15.625	18.2634
C/EBPα [T00108]	373	375	0	CAA	15.625	18.2634
C/EBPα [T00108]	391	393	0	CAA	15.625	18.2634
C/EBPα [T00108]	398	400	0	TTG	15.625	18.2634
C/EBPα [T00108]	415	417	0	TTG	15.625	18.2634
C/EBPα [T00108]	467	469	0	TTG	15.625	18.2634
C/EBPα [T00108]	471	473	0	TTG	15.625	18.2634
C/EBPα [T00108]	480	482	0	TTG	15.625	18.2634
C/EBPα [T00108]	489	491	0	TTG	15.625	18.2634
C/EBPα [T00108]	494	496	0	TTG	15.625	18.2634
C/EBPα [T00108]	499	501	0	TTG	15.625	18.2634
C/EBPα [T00108]	504	506	0	TTG	15.625	18.2634
C/EBPα [T00108]	507	509	0	TTG	15.625	18.2634
C/EBPα [T00108]	554	556	0	TTG	15.625	18.2634
C/EBPα [T00108]	573	575	0	CAA	15.625	18.2634
C/EBPα [T00108]	586	588	0	TTG	15.625	18.2634
C/EBPα [T00108]	604	606	0	CAA	15.625	18.2634
C/EBPα [T00108]	650	652	0	CAA	15.625	18.2634
C/EBPα [T00108]	659	661	0	CAA	15.625	18.2634
C/EBPα [T00108]	665	667	0	TTG	15.625	18.2634
C/EBPα [T00108]	673	675	0	CAA	15.625	18.2634
C/EBPα [T00108]	693	695	0	CAA	15.625	18.2634
C/EBPα [T00108]	698	700	0	TTG	15.625	18.2634
C/EBPα [T00108]	738	740	0	CAA	15.625	18.2634

C/EBPα [T00108]	750	752	0	TTG	15.625	18.2634
C/EBPα [T00108]	813	815	0	CAA	15.625	18.2634
C/EBPα [T00108]	816	818	0	TTG	15.625	18.2634
C/EBPα [T00108]	859	861	0	TTG	15.625	18.2634
C/EBPα [T00108]	917	919	0	TTG	15.625	18.2634
C/EBPα [T00108]	947	949	0	CAA	15.625	18.2634
C/EBPα [T00108]	962	964	0	TTG	15.625	18.2634
C/EBPβ [T00017]	18	22	4.211117	CGTGT	1.95312	1.69217
C/EBPβ [T00017]	58	62	2.100568	ACAGT	1.95312	2.09573
C/EBPβ [T00017]	97	101	2.980795	GGTGT	0.97656	0.6208
C/EBPβ [T00017]	118	122	2.460644	ACATA	2.92969	3.89193
C/EBPβ [T00017]	130	134	2.100568	ACAGT	1.95312	2.09573
C/EBPβ [T00017]	138	142	1.050284	ACAAT	1.95312	2.82176
C/EBPβ [T00017]	193	197	3.33089	ACAGC	2.92969	3.62307
C/EBPβ [T00017]	198	202	2.100568	ACAGT	1.95312	2.09573
C/EBPβ [T00017]	247	251	4.561212	ACAGA	1.95312	1.75703
C/EBPβ [T00017]	327	331	1.050284	ATTGT	1.95312	2.82176
C/EBPβ [T00017]	375	379	4.211117	ACACA	1.95312	1.69217
C/EBPβ [T00017]	377	381	2.460644	ACATA	2.92969	3.89193
C/EBPβ [T00017]	393	397	4.211117	ACACA	1.95312	1.69217
C/EBPβ [T00017]	395	399	0	ACATT	0.97656	1.76092
C/EBPβ [T00017]	397	401	1.050284	ATTGT	1.95312	2.82176
C/EBPβ [T00017]	405	409	2.460644	TATGT	2.92969	3.89193
C/EBPβ [T00017]	414	418	3.510928	CTTGT	2.92969	3.62307
C/EBPβ [T00017]	466	470	3.510928	TTTGT	2.92969	3.62307

C/EBPβ [T00017]	479	483	3.510928	TTTGT	2.92969	3.62307
C/EBPβ [T00017]	488	492	3.510928	TTTGT	2.92969	3.62307
C/EBPβ [T00017]	493	497	3.510928	TTTGT	2.92969	3.62307
C/EBPβ [T00017]	498	502	3.510928	TTTGT	2.92969	3.62307
C/EBPβ [T00017]	503	507	3.510928	TTTGT	2.92969	3.62307
C/EBPβ [T00017]	512	516	4.561212	ACAGG	1.95312	1.75703
C/EBPβ [T00017]	522	526	4.561212	TCTGT	1.95312	1.75703
C/EBPβ [T00017]	536	540	3.33089	GCTGT	2.92969	3.62307
C/EBPβ [T00017]	553	557	3.510928	TTTGT	2.92969	3.62307
C/EBPβ [T00017]	623	627	2.460644	CATGT	2.92969	3.89193
C/EBPβ [T00017]	725	729	2.100568	ACTGT	1.95312	2.09573
C/EBPβ [T00017]	749	753	2.280606	GTTGT	2.92969	3.89193
C/EBPβ [T00017]	807	811	2.460644	ACATG	2.92969	3.89193
C/EBPβ [T00017]	812	816	1.050284	ACAAT	1.95312	2.82176
C/EBPβ [T00017]	815	819	1.050284	ATTGT	1.95312	2.82176
C/EBPβ [T00017]	817	821	4.211117	TGTGT	1.95312	1.69217
C/EBPβ [T00017]	894	898	4.211117	ACACA	1.95312	1.69217
C/EBPβ [T00017]	896	900	2.460644	ACATA	2.92969	3.89193
C/EBPβ [T00017]	899	903	2.460644	TATGT	2.92969	3.89193
C/EBPβ [T00017]	936	940	4.561212	CCTGT	1.95312	1.75703
C/EBPβ [T00017]	961	965	1.050284	ATTGT	1.95312	2.82176
C/EBPβ [T00017]	972	976	2.460644	TATGT	2.92969	3.89193
C/EBPβ [T00017]	978	982	4.561212	TCTGT	1.95312	1.75703
C/EBPβ [T00459]	5	7	0	TTG	15.625	18.2634
C/EBPβ [T00459]	8	10	0	CAA	15.625	18.2634

C/EBPβ [T00459]	74	76	0	CAA	15.625	18.2634
C/EBPβ [T00459]	78	80	0	CAA	15.625	18.2634
C/EBPβ [T00459]	139	141	0	CAA	15.625	18.2634
C/EBPβ [T00459]	164	166	0	TTG	15.625	18.2634
C/EBPβ [T00459]	259	261	0	CAA	15.625	18.2634
C/EBPβ [T00459]	307	309	0	CAA	15.625	18.2634
C/EBPβ [T00459]	328	330	0	TTG	15.625	18.2634
C/EBPβ [T00459]	350	352	0	TTG	15.625	18.2634
C/EBPβ [T00459]	363	365	0	TTG	15.625	18.2634
C/EBPβ [T00459]	373	375	0	CAA	15.625	18.2634
C/EBPβ [T00459]	391	393	0	CAA	15.625	18.2634
C/EBPβ [T00459]	398	400	0	TTG	15.625	18.2634
C/EBPβ [T00459]	415	417	0	TTG	15.625	18.2634
C/EBPβ [T00459]	467	469	0	TTG	15.625	18.2634
C/EBPβ [T00459]	471	473	0	TTG	15.625	18.2634
C/EBPβ [T00459]	480	482	0	TTG	15.625	18.2634
C/EBPβ [T00459]	489	491	0	TTG	15.625	18.2634
C/EBPβ [T00459]	494	496	0	TTG	15.625	18.2634
C/EBPβ [T00459]	499	501	0	TTG	15.625	18.2634
C/EBPβ [T00459]	504	506	0	TTG	15.625	18.2634
C/EBPβ [T00459]	507	509	0	TTG	15.625	18.2634
C/EBPβ [T00459]	554	556	0	TTG	15.625	18.2634
C/EBPβ [T00459]	573	575	0	CAA	15.625	18.2634
C/EBPβ [T00459]	586	588	0	TTG	15.625	18.2634
C/EBPβ [T00459]	604	606	0	CAA	15.625	18.2634

C/EBPβ [T00459]	650	652	0	CAA	15.625	18.2634
C/EBPβ [T00459]	659	661	0	CAA	15.625	18.2634
C/EBPβ [T00459]	665	667	0	TTG	15.625	18.2634
C/EBPβ [T00459]	673	675	0	CAA	15.625	18.2634
C/EBPβ [T00459]	693	695	0	CAA	15.625	18.2634
C/EBPβ [T00459]	698	700	0	TTG	15.625	18.2634
C/EBPβ [T00459]	738	740	0	CAA	15.625	18.2634
C/EBPβ [T00459]	750	752	0	TTG	15.625	18.2634
C/EBPβ [T00459]	813	815	0	CAA	15.625	18.2634
C/EBPβ [T00459]	816	818	0	TTG	15.625	18.2634
C/EBPβ [T00459]	859	861	0	TTG	15.625	18.2634
C/EBPβ [T00459]	917	919	0	TTG	15.625	18.2634
C/EBPβ [T00459]	947	949	0	CAA	15.625	18.2634
C/EBPβ [T00459]	962	964	0	TTG	15.625	18.2634
C/EBPβ [T00581]	4	10	4.018292	CTTGCAA	0.36621	0.425
C/EBPβ [T00581]	5	11	2.809597	TTGCAAT	0.18311	0.2651
C/EBPβ [T00581]	363	369	3.71163	TTGCTAT	0.42725	0.49093
C/EBPβ [T00581]	369	375	1.804066	TGGGCAA	0.30518	0.25219
C/EBPβ [T00581]	586	592	6.263409	TTGCCTG	0.12207	0.07468
CP2 [T00152]	125	132	13.249285	CTGGTACA	0.36621	0.18906
CP2 [T00152]	343	350	12.549128	CTGGCTCT	0.27466	0.16666
CP2 [T00152]	378	385	4.144927	CATACCAG	0.09155	0.05896
CP2 [T00152]	533	540	12.549128	CTGGCTGT	0.27466	0.16666
CP2 [T00152]	566	573	11.78931	CTGGCCTC	0.12207	0.04869
CP2 [T00152]	610	617	8.214579	CTGGGTTT	0.35095	0.15773

CP2 [T00152]	767	774	7.531367	CTGGGAGG	0.13733	0.05611
GATA-1 [T00267]	698	705	6.590036	TTGGATAA	0.04578	0.08525
GATA-1 [T00267]	827	834	1.204329	TTATCGGC	0.01526	0.0115
GATA-1 [T00305]	698	705	4.723851	TTGGATAA	0.16785	0.27481
GATA-1 [T00305]	827	834	3.712083	TTATCGGC	0.03052	0.03103
GATA-1 [T00306]	114	119	1.174121	TATCAC	0.48828	0.65506
GATA-1 [T00306]	133	138	1.174121	GTGATA	0.48828	0.65506
GATA-1 [T00306]	254	259	0.298794	TATCTC	0.73242	1.50427
GATA-1 [T00306]	426	431	0.469533	TATCTT	0.73242	1.50427
GATA-1 [T00306]	699	704	2.76306	TGGATA	0.48828	0.65506
GATA-1 [T00306]	821	826	2.76306	TGGATA	0.48828	0.65506
GATA-1 [T00306]	828	833	2.250843	TATCGG	1.46484	1.47715
GATA-1 [T00306]	882	887	2.76306	TATCGA	0.48828	0.65506
GATA-1 [T00306]	976	981	0	TATCTG	0.24414	0.33435
GATA-2 [T01302]	110	117	8.983724	CGGCTATC	0.18311	0.21354
GATA-2 [T01302]	135	142	12.627873	GATACAAT	0.36621	0.5447
GATA-2 [T01302]	701	708	11.858188	GATAATCT	0.27466	0.49807
GATA-2 [T01302]	823	830	13.806911	GATATTAT	0.36621	0.537
GATA-2 [T01302]	824	831	6.518613	ATATTATC	0.18311	0.35322
GATA-2 [T01302]	878	885	12.627873	TTTATATC	0.36621	0.5447
GATA-3 [T00311]	251	258	5.011765	AAGTATCT	0.10681	0.17988
GATA-3 [T00311]	423	430	4.331086	AAATATCT	0.07629	0.16485
GATA-3 [T00311]	973	980	5.011765	ATGTATCT	0.10681	0.17988
GR [T00333]	20	22	0	TGT	15.625	18.2634
GR [T00333]	58	60	0	ACA	15.625	18.2634

GR [T00333]	99	101	0	TGT	15.625	18.2634
GR [T00333]	118	120	0	ACA	15.625	18.2634
GR [T00333]	130	132	0	ACA	15.625	18.2634
GR [T00333]	138	140	0	ACA	15.625	18.2634
GR [T00333]	193	195	0	ACA	15.625	18.2634
GR [T00333]	198	200	0	ACA	15.625	18.2634
GR [T00333]	247	249	0	ACA	15.625	18.2634
GR [T00333]	329	331	0	TGT	15.625	18.2634
GR [T00333]	375	377	0	ACA	15.625	18.2634
GR [T00333]	377	379	0	ACA	15.625	18.2634
GR [T00333]	393	395	0	ACA	15.625	18.2634
GR [T00333]	395	397	0	ACA	15.625	18.2634
GR [T00333]	399	401	0	TGT	15.625	18.2634
GR [T00333]	407	409	0	TGT	15.625	18.2634
GR [T00333]	416	418	0	TGT	15.625	18.2634
GR [T00333]	468	470	0	TGT	15.625	18.2634
GR [T00333]	481	483	0	TGT	15.625	18.2634
GR [T00333]	490	492	0	TGT	15.625	18.2634
GR [T00333]	495	497	0	TGT	15.625	18.2634
GR [T00333]	500	502	0	TGT	15.625	18.2634
GR [T00333]	505	507	0	TGT	15.625	18.2634
GR [T00333]	512	514	0	ACA	15.625	18.2634
GR [T00333]	524	526	0	TGT	15.625	18.2634
GR [T00333]	538	540	0	TGT	15.625	18.2634
GR [T00333]	555	557	0	TGT	15.625	18.2634

GR [T00333]	625	627	0	TGT	15.625	18.2634
GR [T00333]	727	729	0	TGT	15.625	18.2634
GR [T00333]	751	753	0	TGT	15.625	18.2634
GR [T00333]	807	809	0	ACA	15.625	18.2634
GR [T00333]	812	814	0	ACA	15.625	18.2634
GR [T00333]	817	819	0	TGT	15.625	18.2634
GR [T00333]	819	821	0	TGT	15.625	18.2634
GR [T00333]	894	896	0	ACA	15.625	18.2634
GR [T00333]	896	898	0	ACA	15.625	18.2634
GR [T00333]	901	903	0	TGT	15.625	18.2634
GR [T00333]	938	940	0	TGT	15.625	18.2634
GR [T00333]	963	965	0	TGT	15.625	18.2634
GR [T00333]	974	976	0	TGT	15.625	18.2634
GR [T00333]	980	982	0	TGT	15.625	18.2634
GR [T00333]	996	998	0	ACA	15.625	18.2634
GR [T00335]	234	239	0	AGAACT	0.24414	0.33435
GR [T00335]	517	522	6.726303	GGTTCT	0.48828	0.4118
GR [T00335]	544	549	0	AGAACT	0.24414	0.33435
MyoD [T00526]	194	201	12.73119	CAGCACAG	0.2594	0.20736
MyoD [T00526]	383	390	2.345634	CAGCAGCC	0.12207	0.05525
MyoD [T00526]	605	612	11.00374	AAGTGCTG	0.16785	0.11647
Nrf2:MafK [T05666]	58	64	5.316619	ACAGTAG	0.48828	0.46583
Nrf2:MafK [T05666]	178	184	1.541262	TCAGGAT	0.48828	0.44145
Nrf2:MafK [T05666]	193	199	5.316619	ACAGCAC	0.48828	0.46583
Nrf2:MafK [T05666]	247	253	5.316619	ACAGAAG	0.48828	0.46583

Nrf2:MafK [T05666]	340	346	8.033921	TTTCTGG	0.73242	0.65091
Nrf2:MafK [T05666]	382	388	9.33358	CCAGCAG	0.97656	0.53743
Nrf2:MafK [T05666]	578	584	0	TCAGAAA	0.36621	0.5362
Nrf2:MafK [T05666]	607	613	9.33358	GTGCTGG	0.97656	0.53743
Nrf2:MafK [T05666]	934	940	5.558222	TTCCTGT	0.48828	0.46583
Nrf2:MafK [T05666]	940	946	1.299659	TCAGTAC	0.48828	0.44145
p53 [T00671]	281	287	5.253995	AATGCCC	0.12207	0.05784
p53 [T00671]	287	293	3.057625	CACGCCC	0.12207	0.03427
p53 [T00671]	370	376	5.084756	GGGCAAC	0.24414	0.06544
p53 [T00671]	385	391	7.965283	GCAGCCC	0.48828	0.10994
p53 [T00671]	527	533	8.842914	ATAGCCC	0.36621	0.11232
Sp1 [T00755]	286	294	6.497596	CCACGCCCT	0.03815	0.00765
Sp1 [T01228]	285	295	13.898109	CCCACGCCCTA	0.06652	0.01606
SRF [T00763]	692	701	12.836461	CCAAATTTGG	0.06008	0.07257
SRF [T00766]	690	701	13.481114	TTCCAAATTTGG	0.03552	0.04218
SRF [T00766]	692	703	13.341643	CCAAATTTGGAT	0.03552	0.04218
YY1 [T00278]	108	118	14.35951	TGCGGCTATCA	0.08011	0.02862
YY1 [T00865]	10	15	0.700325	ATGGAC	0.73242	0.87617
YY1 [T00865]	141	146	0	ATGGAG	0.24414	0.20504
YY1 [T00865]	207	212	2.965889	TCCCAT	0.73242	0.59472
YY1 [T00865]	261	266	0.350162	ATGGAA	0.73242	0.87617
YY1 [T00865]	368	373	3.316052	ATGGGC	0.73242	0.77254
YY1 [T00865]	430	435	0.350162	TTCCAT	0.73242	0.87617
YY1 [T00865]	838	843	3.14097	ACCCAT	0.73242	0.59472
YY1 [T00915]	10	15	1.17887	ATGGAC	1.2207	0.98802

YY1 [T00915]	141	146	0.781798	ATGGAG	1.2207	0.98802
YY1 [T00915]	207	212	2.177379	TCCCAT	0.97656	1.05597
YY1 [T00915]	261	266	1.708301	ATGGAA	1.2207	1.10967
YY1 [T00915]	368	373	1.647949	ATGGGC	1.2207	1.10967
YY1 [T00915]	430	435	1.708301	TTCCAT	1.2207	1.10967
YY1 [T00915]	838	843	2.045022	ACCCAT	0.97656	1.05597
YY1 [T04970]	187	195	5.873209	GCCTAGACA	0.18311	0.12783
YY1 [T04970]	506	514	7.047851	GTTGAGACA	0.15259	0.11378
YY1 [T04970]	806	814	11.458499	CACATGACA	0.27466	0.19711
YY1 [T04970]	938	946	10.571776	TGTCAGTAC	0.33569	0.24819

Supplementary Table S2. List of putative transcription factor binding sites detected in mouse FTH1 upstream 1.0-kb predicted with cut-off matrix similarity > 0.85 in TRANSCFAC.

factor name	position	strand	core match	matrix match	sequence (always the (+)-strand is shown)
AP-1	100	(+)	0.906	0.895	tgTGATTaatg
AP-1	101	(-)	0.964	0.884	gTGATTaat
AP-1	544	(-)	0.908	0.854	tagaACTCAct
AP-1	596	(+)	0.983	0.953	tCTGACtccca
AP-1	596	(+)	1	0.932	tcTGACTccca
AP-1	596	(+)	1	0.951	tcTGACTccca
AP-1	596	(+)	1	0.916	tcTGACTccca
AP-1	758	(-)	0.903	0.894	tttaATTCAcc
AP-1	791	(-)	0.903	0.896	agttATTCAtt
AP-1	792	(+)	0.955	0.878	gttATTCAt
AP-1	809	(+)	0.975	0.907	cATGACaattg
AP-1	809	(+)	0.967	0.872	caTGACAattg
AP-1	809	(+)	0.936	0.871	caTGACAattg
AP-1	935	(-)	0.983	0.933	ttcctGTCAGt
AP-1	935	(-)	0.967	0.92	ttccTGTCAgt
AP-1	935	(-)	0.936	0.868	ttccTGTCAgt
AP-1	935	(-)	0.935	0.855	ttccTGTCAgt
AP-1	947	(-)	0.975	0.919	ccaaaGTCATa
AP-1	947	(-)	1	0.9	ccaaAGTCAta
AP-1	947	(-)	1	0.925	ccaaAGTCAta
AP-1	947	(-)	1	0.89	ccaaAGTCAta
C/EBP	1	(-)	1	0.954	gcatcttGCAATgg

C/EBP	3	(+)	0.984	0.881	atCTTGCaatggac
C/EBP	98	(+)	0.94	0.937	ggTGTGAttaatg
C/EBP	132	(-)	0.949	0.924	cagtgatACAATgg
C/EBP	146	(+)	0.885	0.852	agTCTGAgcacta
C/EBP	147	(-)	0.876	0.874	gtctgaGCACTag
C/EBP	162	(+)	0.961	0.921	caTTTGAgaatat
C/EBP	242	(-)	0.889	0.893	aactaaACAGAag
C/EBP	252	(-)	0.944	0.909	aagtatcTCAATgg
C/EBP	326	(+)	0.889	0.86	taATTGTgcataa
C/EBP	326	(+)	0.949	0.866	taATTGTgcataaa
C/EBP	327	(-)	0.917	0.904	aattgtGCATAaa
C/EBP	328	(+)	0.952	0.929	atTGTGCataaac
C/EBP	361	(+)	0.984	0.875	tcCTTGCtatgggc
C/EBP	366	(+)	0.945	0.894	gcTATGGgcaaca
C/EBP	366	(-)	0.987	0.886	gctatggGCAACac
C/EBP	404	(+)	0.91	0.928	tcTATGTtaacct
C/EBP	413	(+)	0.933	0.936	acCTTGTttaaaaa
C/EBP	469	(+)	1	0.887	tgTTTGGtttttt
C/EBP	478	(+)	0.965	0.855	ttTTTGTtttttt
C/EBP	487	(+)	0.965	0.856	ttTTTGTtttgtt
C/EBP	502	(+)	0.965	0.946	gtTTTGTtgagac
C/EBP	502	(+)	0.933	0.859	gtTTTGTtgagaca
C/EBP	505	(+)	0.931	0.904	ttGTTGAgacaggg
C/EBP	521	(+)	0.889	0.893	tcTCTGTatagcc
C/EBP	552	(+)	0.965	0.938	acTTTGTagatca

C/EBP	584	(+)	0.984	0.855	aaTTTGCctgcctc
C/EBP	643	(+)	0.904	0.869	taAGTGGccaaac
C/EBP	644	(-)	1	0.935	aagtggCCAAAcc
C/EBP	686	(-)	0.936	0.905	ccctattCCAAAtt
C/EBP	687	(-)	1	0.875	cctattCCAAAtt
C/EBP	696	(+)	1	0.973	aaTTTGGataatc
C/EBP	696	(+)	0.936	0.942	aaTTTGGataatct
C/EBP	800	(-)	0.979	0.923	ttttagCCACAtg
C/EBP	814	(+)	0.949	0.852	caATTGTgtggata
C/EBP	816	(+)	0.944	0.894	atTGTGTggatat
C/EBP	818	(+)	0.979	0.962	tgTGTGGatatta
C/EBP	845	(-)	0.941	0.855	tcttcCTTCCctaatttg
C/EBP	887	(-)	0.979	0.947	gagttcCCACAca
C/EBP	960	(+)	0.949	0.915	ttATTGTaatatat
C/EBP	977	(+)	0.889	0.862	taTCTGTgcatat
C/EBP	978	(-)	0.917	0.899	atctgtGCATAtg
C/EBP	979	(+)	0.952	0.942	tcTGTGCatatgc
C/EBPα	1	(-)	1	0.916	gcatcttGCAATgg
C/EBPα	132	(-)	0.929	0.876	cagtgatACAATgg
C/EBPα	161	(+)	0.872	0.877	tcATTTGagaatat
C/EBPα	161	(-)	0.858	0.879	tcatttgAGAATat
C/EBPα	213	(+)	0.875	0.891	tcTTTAAataaaaa
C/EBPα	213	(-)	0.872	0.888	tctttaaATAAAaa
C/EBPα	252	(-)	0.932	0.864	aagtatcTCAATgg
C/EBPα	326	(+)	0.929	0.856	taATTGTgcataaa

C/EBPα	366	(-)	0.979	0.893	gctatggGCAACac
C/EBPα	413	(+)	0.903	0.916	acCTTGTttaaaaa
C/EBPα	413	(-)	0.875	0.889	accttgtTTAAAaa
C/EBPα	416	(-)	0.858	0.88	ttgtttaAAAATat
C/EBPα	505	(+)	0.91	0.861	ttGTTGAgacaggg
C/EBPα	575	(-)	0.929	0.854	aaactcaGAAATtt
C/EBPα	643	(-)	0.92	0.859	taagtggCCAAAcc
C/EBPα	661	(-)	0.908	0.917	aacttttGAAACtc
C/EBPα	681	(+)	0.929	0.858	atATTCCctattcc
C/EBPα	686	(-)	0.92	0.855	ccctattCCAAAtt
C/EBPα	688	(+)	0.929	0.855	ctATTCCaaatttg
C/EBPα	696	(+)	0.92	0.931	aaTTTGGataatct
C/EBPα	696	(-)	0.895	0.907	aatttggATAATct
C/EBPα	775	(+)	0.858	0.875	gtATTTTtaaatac
C/EBPα	775	(-)	0.861	0.878	gtattttTAAATac
C/EBPα	848	(+)	0.903	0.913	tcCTTCCctaattt
C/EBPα	848	(-)	0.909	0.909	tccttccCTAATtt
C/EBPα	855	(-)	0.929	0.854	ctaatttGAAATgc
C/EBPα	887	(+)	0.908	0.861	gaGTTCCcacacat
C/EBPα	958	(-)	0.966	0.893	ctttattGTAATat
C/EBPα	960	(+)	0.929	0.863	ttATTGTaatatat
C/EBPβ	1	(+)	1	0.931	gcatcttGCAATgg
C/EBPβ	1	(-)	1	0.886	gcatctTGCAAtgg
C/EBPβ	174	(-)	0.981	0.892	tgCTTTCaggatta
C/EBPβ	661	(+)	0.966	0.954	aacttttGAAACtc

C/EBPβ	661	(-)	0.865	0.872	aactttTGAAActc
C/EBPβ	848	(-)	0.95	0.954	tcCTTCCctaattt
C/EBPβ	887	(-)	0.935	0.911	gaGTTCCcacacat
C/EBPβ	958	(+)	0.981	0.897	ctttattGTAATat
CP2	344	(-)	0.974	0.853	CTGGCtcttga
GATA1	100	(+)	0.985	0.965	tgtGATTAat
GATA1	110	(-)	0.993	0.944	gcggcTATCAcata
GATA1	110	(-)	0.96	0.913	gcggCTATCacata
GATA1	110	(-)	0.991	0.954	gcggcTATCAcat
GATA1	112	(-)	1	0.981	ggCTATCaca
GATA1	131	(+)	0.993	0.935	acagTGATAcaatg
GATA1	131	(+)	0.945	0.898	acagtGATACaatg
GATA1	132	(+)	0.991	0.922	cagTGATAcaatg
GATA1	133	(+)	0.993	0.97	agtGATACaa
GATA1	156	(+)	0.983	0.972	ctaGATCAtt
GATA1	156	(-)	0.985	0.96	ctAGATCatt
GATA1	180	(+)	0.985	0.977	cagGATTAgc
GATA1	203	(+)	0.988	0.967	tgaGATCCca
GATA1	203	(-)	0.985	0.964	tgAGATCcca
GATA1	208	(-)	1	0.974	tcCCATCttt
GATA1	250	(-)	1	0.947	agaagTATCTcaat
GATA1	250	(-)	0.945	0.899	agaaGTATCtcaat
GATA1	250	(-)	1	0.936	agaagTATCTcaa
GATA1	252	(-)	0.993	0.975	aaGTATCtca
GATA1	299	(-)	0.988	0.973	ttTCATCctc

GATA1	350	(+)	0.985	0.973	cttGATCTtc
GATA1	350	(-)	0.983	0.972	ctTGATCttc
GATA1	356	(-)	0.988	0.962	ctTCATCctt
GATA1	422	(-)	1	0.941	aaaaaTATCTtcca
GATA1	422	(-)	0.937	0.893	aaaaATATCttcca
GATA1	422	(-)	1	0.939	aaaaaTATCTtcc
GATA1	424	(-)	0.99	0.98	aaATATCttc
GATA1	557	(+)	0.983	0.972	gtaGATCAgg
GATA1	557	(-)	0.985	0.977	gtAGATCagg
GATA1	697	(+)	0.986	0.939	atttGGATAatctt
GATA1	697	(+)	1	0.945	atttgGATAAtctt
GATA1	698	(+)	0.965	0.932	tttGGATAatctt
GATA1	699	(+)	0.988	0.96	ttgGATAAtc
GATA1	699	(+)	1	0.909	ttgGATAAtc
GATA1	699	(+)	1	0.877	ttgGATAAtc
GATA1	702	(-)	0.985	0.96	gaTAATCttt
GATA1	731	(+)	0.987	0.957	ataGATTTca
GATA1	819	(+)	0.986	0.937	gtgtGGATAttatc
GATA1	819	(+)	0.937	0.9	gtgtgGATATtatc
GATA1	820	(+)	0.965	0.901	tgtGGATAttatc
GATA1	821	(+)	0.99	0.974	gtgGATATta
GATA1	824	(-)	0.991	0.946	gatatTATCGgcta
GATA1	824	(-)	1	0.938	gataTTATCGgcta
GATA1	824	(-)	0.965	0.952	gatatTATCGgct
GATA1	826	(-)	0.988	0.979	taTTATCggc

GATA1	826	(-)	1	0.957	taTTATCggc
GATA1	826	(-)	1	0.924	taTTATCggc
GATA1	878	(-)	0.991	0.924	gtttaTATCGagtt
GATA1	878	(-)	0.937	0.896	gtttATATCgagtt
GATA1	878	(-)	0.965	0.912	gtttaTATCGagt
GATA1	880	(-)	0.99	0.974	ttATATCgag
GATA1	917	(+)	0.987	0.961	tttGATTTtt
GATA1	928	(-)	0.988	0.977	tcTCATCttc
GATA1	972	(-)	1	0.938	atatgTATCTgtgc
GATA1	972	(-)	0.945	0.897	atatGTATCtgtgc
GATA1	972	(-)	1	0.946	atatgTATCTgtg
GATA1	974	(-)	0.993	0.97	atGTATCtgt
GATA1	988	(-)	0.993	0.972	atGCATCcca
GATA2	100	(+)	0.971	0.961	tgtGATTAat
GATA2	112	(-)	1	0.996	ggCTATCaca
GATA2	133	(+)	0.995	0.98	agtGATACaa
GATA2	133	(+)	0.931	0.909	agtGATACaa
GATA2	156	(+)	0.971	0.952	ctaGATCAtt
GATA2	156	(-)	0.972	0.958	ctAGATCatt
GATA2	180	(+)	0.971	0.964	cagGATTAgc
GATA2	203	(+)	0.974	0.969	tgaGATCCca
GATA2	203	(-)	0.972	0.968	tgAGATCcca
GATA2	208	(-)	0.987	0.974	tcCCATCttt
GATA2	252	(-)	0.995	0.988	aaGTATCtca
GATA2	299	(-)	0.979	0.967	ttTCATCctc

GATA2	350	(+)	0.972	0.953	cttGATCTtc
GATA2	350	(-)	0.971	0.961	ctTGATCttc
GATA2	356	(-)	0.979	0.966	ctTCATCctt
GATA2	424	(-)	0.994	0.986	aaATATCttc
GATA2	557	(+)	0.971	0.955	gtaGATCAgg
GATA2	557	(-)	0.972	0.963	gtAGATCagg
GATA2	699	(+)	0.992	0.972	ttgGATAAtc
GATA2	699	(+)	1	0.922	ttgGATAAtc
GATA2	702	(-)	0.971	0.96	gaTAATCttt
GATA2	731	(+)	0.972	0.951	ataGATTTca
GATA2	821	(+)	0.994	0.973	gtgGATATta
GATA2	821	(+)	0.954	0.881	gtgGATATta
GATA2	826	(-)	0.992	0.985	taTTATCggc
GATA2	826	(-)	1	0.948	taTTATCggc
GATA2	826	(-)	1	0.943	taTTATCggc
GATA2	880	(-)	0.994	0.968	ttATATCgag
GATA2	880	(-)	0.954	0.893	ttATATCgag
GATA2	917	(+)	0.972	0.952	tttGATTTtt
GATA2	928	(-)	0.979	0.971	tcTCATCttc
GATA2	928	(-)	0.871	0.881	tcTCATCttc
GATA2	974	(-)	0.995	0.982	atGTATCtgt
GATA2	988	(-)	0.982	0.972	atGCATCcca
GATA3	100	(+)	0.925	0.86	tgtGATTAat
GATA3	101	(+)	0.962	0.932	gtGATTAat
GATA3	112	(-)	1	0.969	ggCTATCac

GATA3	133	(+)	0.878	0.86	agtGATACaa
GATA3	133	(+)	0.896	0.86	agtGATACaa
GATA3	134	(+)	0.974	0.951	gtGATACaa
GATA3	156	(-)	0.936	0.929	ctAGATCat
GATA3	156	(+)	0.928	0.871	ctaGATCAtt
GATA3	156	(+)	0.977	0.862	ctaGATCAtt
GATA3	156	(-)	1	0.857	ctAGATCatt
GATA3	157	(+)	0.944	0.917	taGATCAtt
GATA3	181	(+)	0.962	0.933	agGATTAgc
GATA3	203	(-)	0.936	0.913	tgAGATCcc
GATA3	203	(+)	0.915	0.869	tgaGATCCca
GATA3	203	(-)	1	0.868	tgAGATCcca
GATA3	204	(+)	0.933	0.915	gaGATCCca
GATA3	208	(-)	0.969	0.952	tcCCATCtt
GATA3	208	(-)	0.87	0.88	tcCCATCttt
GATA3	252	(-)	0.974	0.949	aaGTATCtc
GATA3	299	(-)	0.954	0.928	ttTCATCct
GATA3	299	(-)	0.917	0.867	ttTCATCctc
GATA3	350	(-)	0.944	0.94	ctTGATCtt
GATA3	350	(-)	0.928	0.878	ctTGATCttc
GATA3	350	(-)	0.977	0.854	ctTGATCttc
GATA3	351	(+)	0.936	0.906	ttGATCTtc
GATA3	356	(-)	0.954	0.945	ctTCATCct
GATA3	424	(-)	0.977	0.95	aaATATCtt
GATA3	424	(-)	0.981	0.872	aaATATCttc

GATA3	557	(-)	0.936	0.905	gtAGATCag
GATA3	557	(+)	0.928	0.872	gtaGATCAgg
GATA3	558	(+)	0.944	0.943	taGATCAgg
GATA3	699	(+)	1	0.892	ttgGATAAtc
GATA3	700	(+)	0.985	0.951	tgGATAAtc
GATA3	702	(-)	0.962	0.935	gaTAATCtt
GATA3	702	(-)	0.925	0.877	gaTAATCttt
GATA3	702	(-)	0.945	0.875	gaTAATCttt
GATA3	731	(+)	0.968	0.943	ataGATTTca
GATA3	732	(+)	0.953	0.931	taGATTTca
GATA3	821	(+)	0.981	0.885	gtgGATATta
GATA3	822	(+)	0.977	0.95	tgGATATta
GATA3	826	(-)	0.985	0.961	taTTATCgg
GATA3	826	(-)	1	0.943	taTTATCggc
GATA3	826	(-)	0.958	0.874	taTTATCggc
GATA3	880	(-)	0.977	0.95	ttATATCga
GATA3	880	(-)	0.981	0.876	ttATATCgag
GATA3	918	(+)	0.953	0.922	ttGATTTtt
GATA3	928	(-)	0.954	0.938	tcTCATCtt
GATA3	928	(-)	0.917	0.905	tcTCATCttc
GATA3	928	(-)	0.932	0.899	tcTCATCttc
GATA3	974	(-)	0.974	0.945	atGTATCtg
GATA3	988	(-)	0.943	0.913	atGCATCcc
GR	10	(+)	0.986	0.94	aatggactgcgTGTGCtag
GR	12	(+)	0.96	0.852	tggactgcgtGTGCTa

GR	126	(-)	0.984	0.87	ctgGTACAgtgatacaatg
GR	194	(-)	0.986	0.961	acaGCACAgtgagatccca
GR	319	(+)	0.986	0.88	aaatatataatTGTGCata
GR	471	(+)	0.875	0.854	tttggttttttTGTTTttt
GR	480	(+)	0.875	0.854	tttgtttttttTGTTTtgt
GR	490	(+)	0.875	0.852	ttgttttgtttTGTTTtgt
GR	528	(+)	0.989	0.939	atagccctggcTGTCCtag
GR	615	(+)	0.986	0.856	gtttaaaggcaTGTGCcac
GR	970	(+)	0.986	0.904	atatatgtatcTGTGCata
MyoD	602	(+)	0.873	0.851	tccCAAGTgctg
MyoD	621	(+)	0.905	0.861	aggCATGTgcca
MyoD	622	(+)	0.851	0.889	ggCATGTgcc
MyoD	762	(-)	1	0.856	attcACCTGgga
MyoD	763	(+)	1	0.921	ttCACCTggg
MyoD	804	(-)	0.905	0.861	agccACATGaca
MyoD	805	(-)	0.851	0.882	gccACATGac
NRF-2	261	(+)	1	0.868	aatGGAAGag
NRF-2	932	(-)	1	0.901	atCTTCCtgt
p53	587	(+)	0.965	0.882	ttgCCTGCct
p53	621	(+)	1	0.87	aggCATGTgc
p53	621	(-)	1	0.922	agGCATGtgc
p53	652	(+)	0.965	0.897	aaaCCTGCca
p53	865	(+)	1	0.91	atgCATGCct
p53	865	(-)	1	0.878	atGCATGcct
Sp1	286	(-)	0.949	0.9	cccACGCCct

Sp1	563	(+)	0.962	0.873	caGGCTGgcc
Sp1	588	(-)	0.972	0.908	tgcCTGCCtc
Sp1	653	(-)	0.972	0.897	aacCTGCCaa
Sp1	866	(-)	0.921	0.852	tgcATGCCtt
Sp1	902	(+)	0.915	0.872	tgtgGGTGGagt
Sp1	903	(+)	0.872	0.854	gtGGGTGgga
SRF	691	(+)	0.99	0.922	ttCCAAAtttggat
SRF	691	(-)	0.99	0.92	ttccaaaTTTGGat
SRF	692	(+)	0.867	0.851	tccaaaTTTGGataa
YY1	3	(-)	1	0.9	atcttgcaATGGActgc
YY1	134	(-)	1	0.922	gtgatacaATGGAgtct
YY1	156	(+)	0.922	0.891	ctagATCATttgagaat
YY1	165	(-)	0.906	0.907	ttgagaatATGCTttca
YY1	205	(+)	0.996	0.984	agatCCCATctttaaat
YY1	254	(-)	1	0.899	gtatctcaATGGAagag
YY1	361	(-)	0.996	0.869	tccttgctATGGGcaac
YY1	428	(+)	1	0.95	atctTCCATataaaccc
YY1	618	(+)	0.904	0.866	taaaGGCATgtgccacc
YY1	792	(+)	0.922	0.898	gttaTTCATtttagcca
YY1	802	(-)	0.92	0.891	ttagccacATGACaatt
YY1	836	(+)	0.996	0.91	taaaCCCATtcttcctt
YY1	857	(-)	0.906	0.875	aatttgaaATGCAtgcc
YY1	892	(+)	0.902	0.86	cccaCACATatgtgggt
YY1	893	(-)	0.902	0.864	ccacacatATGTGggtg
YY1	925	(+)	0.918	0.887	ttctCTCATcttcctgt

YY1	966	(-)	0.906	0.887	taatatatATGTAtctg
YY1	979	(+)	0.906	0.864	tctgTGCATatgcatcc
YY1	980	(-)	0.906	0.879	ctgtgcatATGCAtccc

Supplementary Table S3. List of putative transcription factor binding sites detected in mouse FTH1 upstream 1.0-kb predicted with cut-off Score > 0.85 in TF BIND.

ID	Score	Loc.	Str.	Consensus	Sequence
AP1_C	0.871429	101	(-)	NTGASTCAG	GTGATTAAT
AP1_C	0.85277	597	(-)	NTGASTCAG	CTGACTCCC
AP1_C	0.856268	792	(-)	NTGASTCAG	GTTATTCAT
AP1_Q6	0.872508	596	(+)	NNTGACTCANN	TCTGACTCCCA
CEBP_01	0.883306	404	(+)	NNTKTGGWNANNN	TCTATGTTAACCT
CEBP_01	0.935494	469	(+)	NNTKTGGWNANNN	TGTTTGGTTTTTT
CEBP_01	0.871637	487	(+)	NNTKTGGWNANNN	TTTTTGTTTTGTT
CEBP_01	0.914749	687	(-)	NNTKTGGWNANNN	CCTATTCCAAATT
CEBP_01	0.947488	696	(+)	NNTKTGGWNANNN	AATTTGGATAATC
CEBP_01	0.929011	818	(+)	NNTKTGGWNANNN	TGTGTGGATATTA
CEBP_01	0.926742	887	(-)	NNTKTGGWNANNN	GAGTTCCCACACA
CEBP_01	0.9141	979	(+)	NNTKTGGWNANNN	TCTGTGCATATGC
CEBP_Q2	0.859404	1	(-)	NNNTTGCNNAANNN	GCATCTTGCAATGG
CEBP_Q2	0.860876	413	(+)	NNNTTGCNNAANNN	ACCTTGTTTAAAAA
CEBP_Q2	0.853147	696	(+)	NNNTTGCNNAANNN	AATTTGGATAATCT
CEBPA_01	0.876809	696	(+)	NNATTRCNNAANNN	AATTTGGATAATCT
CEBPB_01	0.867509	733	(-)	RNRTKNNGMAAKNN	AGATTTCAACTTTA
CEBPB_01	0.857355	1	(+)	RNRTKNNGMAAKNN	GCATCTTGCAATGG
CEBPB_01	0.855869	505	(+)	RNRTKNNGMAAKNN	TTGTTGAGACAGGG

CEBPB_01	0.854631	661	(+)	RNRTKNNGMAAKNN	AACTTTTGAAACTC
CP2_01	0.914666	363	(-)	GCNMNAMCMAG	CTTGCTATGGG
CP2_01	0.886364	834	(+)	GCNMNAMCMAG	GCTAAACCCAT
CP2_01	0.867496	344	(-)	GCNMNAMCMAG	CTGGCTCTTGA
CP2_01	0.858919	648	(+)	GCNMNAMCMAG	GGCCAAACCTG
CP2_01	0.856775	72	(+)	GCNMNAMCMAG	AGCCAACCAAG
GATA1_01	0.90079	112	(-)	SNNGATNNNN	GGCTATCACA
GATA1_01	0.8692	252	(-)	SNNGATNNNN	AAGTATCTCA
GATA1_01	0.875617	424	(-)	SNNGATNNNN	AAATATCTTC
GATA1_01	0.857354	826	(-)	SNNGATNNNN	TATTATCGGC
GATA1_03	0.863792	926	(-)	RNSNNGATAANNGN	TCTCTCATCTTCCT
GATA1_04	0.887255	110	(-)	NNCWGATARNNNN	GCGGCTATCACAT
GATA2_01	0.943166	112	(-)	NNNGATRNNN	GGCTATCACA
GATA2_01	0.913848	252	(-)	NNNGATRNNN	AAGTATCTCA
GATA2_01	0.908886	424	(-)	NNNGATRNNN	AAATATCTTC
GATA2_01	0.869193	826	(-)	NNNGATRNNN	TATTATCGGC
GATA2_01	0.85972	988	(-)	NNNGATRNNN	ATGCATCCCA
GATA3_01	0.87284	112	(-)	NNGATARNG	GGCTATCAC
GATA3_01	0.894107	208	(-)	NNGATARNG	TCCCATCTT
GATA3_01	0.863093	826	(-)	NNGATARNG	TATTATCGG
GATA3_01	0.865751	928	(-)	NNGATARNG	TCTCATCTT
GR_Q6	0.879464	194	(-)	NNNNNNCNNTNTGTNCTNN	ACAGCACAGTGAGATCCCA

MYOD_01	0.897047	621	(+)	SRACAGGTGKYG	AGGCATGTGCCA
MYOD_01	0.850197	804	(-)	SRACAGGTGKYG	AGCCACATGACA
MYOD_Q6	0.874152	622	(+)	NNCANCTGNY	GGCATGTGCC
NRF2_01	0.925388	932	(-)	ACCGGAAGNS	ATCTTCCTGT
NRF2_01	0.853924	261	(+)	ACCGGAAGNS	AATGGAAGAG
P53_02	0.890387	564	(-)	NGRCWTGYCY	AGGCTGGCCT
P53_02	0.885991	564	(+)	NGRCWTGYCY	AGGCTGGCCT
P53_02	0.867233	583	(+)	NGRCWTGYCY	AAATTTGCCT
P53_02	0.899766	621	(-)	NGRCWTGYCY	AGGCATGTGC
P53_02	0.856975	621	(+)	NGRCWTGYCY	AGGCATGTGC
P53_02	0.872802	652	(+)	NGRCWTGYCY	AAACCTGCCA
P53_02	0.862837	652	(-)	NGRCWTGYCY	AAACCTGCCA
P53_02	0.91823	865	(+)	NGRCWTGYCY	ATGCATGCCT
P53_02	0.866061	865	(-)	NGRCWTGYCY	ATGCATGCCT
SP1_01	0.855323	280	(-)	GRGGCRGGGW	AGAATGCCCA
SP1_01	0.908834	588	(-)	GRGGCRGGGW	TGCCTGCCTC
SP1_01	0.85051	653	(-)	GRGGCRGGGW	AACCTGCCAA
SP1_01	0.855606	866	(-)	GRGGCRGGGW	TGCATGCCTT
SP1_01	0.864666	903	(+)	GRGGCRGGGW	GTGGGTGGGA
SRF_C	0.870696	689	(-)	NCCWTATATGGNCWN	TATTCCAAATTTGGA
SRF_C	0.87052	692	(+)	NCCWTATATGGNCWN	TCCAAATTTGGATAA
SRF_Q6	0.909975	691	(+)	GNCCAWATAWGGMN	TTCCAAATTTGGAT

SRF_Q6	0.902156	691	(-)	GNCCAWATAWGGMN	TTCCAAATTTGGAT
YY1_01	0.861402	134	(-)	NNNNNCCATNTWNNNWN	GTGATACAATGGAGTCT
YY1_01	0.854849	205	(+)	NNNNNCCATNTWNNNWN	AGATCCCATCTTTAAAT

Supplementary Table S4. Analysis of the correlation between the iron content in the mouse liver and the analyzed genes in microarray

Gene	RHO	*p*-value	Regulation	Functions	Reference
Scd1	-0.57471	0.002321	Down	▪ Fatty Acyl-CoA Biosynthesis<br>▪ Regulation of cholesterol biosynthesis by SREBP	[1,2]
Acsl5	-0.659	0.002321	n.s.	▪ Fatty Acyl-CoA Biosynthesis<br>▪ Regulation of lipid droplet	[3,4]
Leptin	0.608648	0.000754	n.s.	▪ AMPK Signaling<br>▪ Adipocytokine signaling pathway	[5]
ASXL1	-0.54461	0.003154	Up	▪ Transcriptional regulator of adipogenesis, acting to repress PPARγ adipocyte differentiation	[6]
GATA2	-0.54351	0.003225	n.s.	▪ Regulation of differentiation of brown adipocytes<br>▪ Inhibitor of PPARγ	[7]

All data was given in a study by McLachlan, S., et al. [8]. Abbreviation: Scd1; Stearoyl-CoA desaturase-1, Acsl5; Acyl-CoA Synthetase Long Chain Family Member 5, ASXL1; Additional Sex Comb-Like Protein 1, Gata2; GATA Binding Protein

Supplementary Figure S1. The standard curve for measurement of total glutathione (tGSH) contents

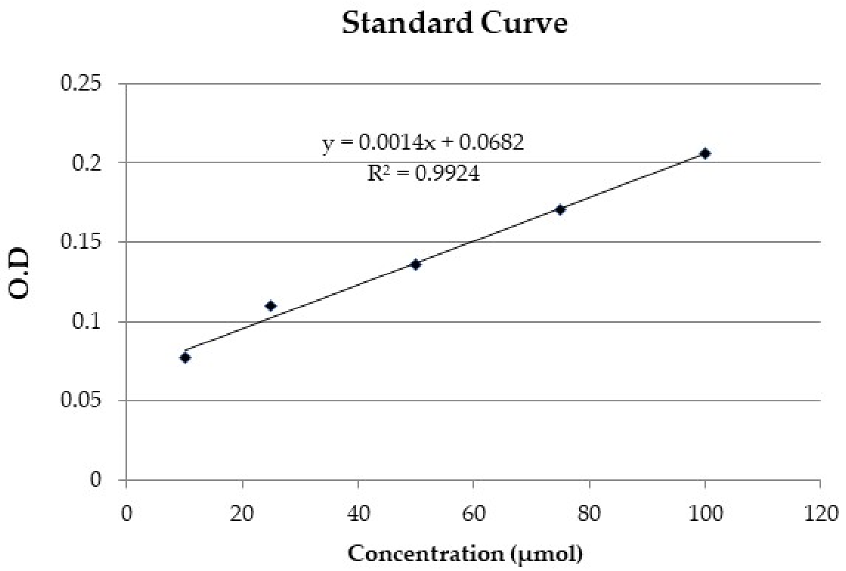

Reference

1. Ntambi, J.M.; Miyazaki, M.; Stoehr, J.P.; Lan, H.; Kendziorski, C.M.; Yandell, B.S.; Song, Y.; Cohen, P.; Friedman, J.M.; Attie, A.D. Loss of stearoyl–CoA desaturase-1 function protects mice against adiposity. *Proceedings of the National Academy of Sciences* **2002**, *99*, 11482-11486.
2. Sekiya, M.; Yahagi, N.; Matsuzaka, T.; Takeuchi, Y.; Nakagawa, Y.; Takahashi, H.; Okazaki, H.; Iizuka, Y.; Ohashi, K.; Gotoda, T. SREBP-1-independent regulation of lipogenic gene expression in adipocytes. *Journal of lipid research* **2007**, *48*, 1581-1591.
3. Bowman, T.A.; O'Keeffe, K.R.; D'Aquila, T.; Yan, Q.W.; Griffin, J.D.; Killion, E.A.; Salter, D.M.; Mashek, D.G.; Buhman, K.K.; Greenberg, A.S. Acyl CoA synthetase 5 (ACSL5) ablation in mice increases energy expenditure and insulin sensitivity and delays fat absorption. *Molecular metabolism* **2016**, *5*, 210-220.
4. Zhang, M.; Liu, Y.; Shi, H.; Guo, M.; Chai, M.; He, Q.; Yan, M.; Cao, D.; Zhao, L.; Cai, H. Evolutionary and expression analyses of soybean basic Leucine zipper transcription factor family. *BMC genomics* **2018**, *19*, 1-14.
5. Lage, R.; Diéguez, C.; Vidal-Puig, A.; López, M. AMPK: a metabolic gauge regulating whole-body energy homeostasis. *Trends in molecular medicine* **2008**, *14*, 539-549.
6. Park, U.-H.; Yoon, S.K.; Park, T.; Kim, E.-J.; Um, S.-J. Additional sex comb-like (ASXL) proteins 1 and 2 play opposite roles in adipogenesis via reciprocal regulation of peroxisome proliferator-activated receptor γ. *Journal of Biological Chemistry* **2011**, *286*, 1354-1363.
7. Schupp, M.; Cristancho, A.G.; Lefterova, M.I.; Hanniman, E.A.; Briggs, E.R.; Steger, D.J.; Qatanani, M.; Curtin, J.C.; Schug, J.; Ochsner, S.A. Re-expression of GATA2 cooperates with peroxisome proliferator-activated receptor-γ depletion to revert the adipocyte phenotype. *Journal of Biological Chemistry* **2009**, *284*, 9458-9464.
8. McLachlan, S.; Lee, S.-M.; Steele, T.M.; Hawthorne, P.L.; Zapala, M.A.; Eskin, E.; Schork, N.J.; Anderson, G.J.; Vulpe, C.D. In silico QTL mapping of basal liver iron levels in inbred mouse strains. *Physiological genomics* **2011**, *43*, 136-147.
